# Resistance to Multiple Tuber Diseases Expressed in Somaclonal Variants of the Potato Cultivar Russet Burbank

**DOI:** 10.1155/2014/417697

**Published:** 2014-01-12

**Authors:** Tamilarasan Thangavel, Robert Steven Tegg, Calum Rae Wilson

**Affiliations:** Tasmanian Institute of Agriculture, University of Tasmania, New Town Research Laboratories, 13 St. Johns' Avenue, New Town, TAS 7008, Australia

## Abstract

Multiple disease resistance is an aim of many plant breeding programs. Previously, novel somatic cell selection was used to generate potato variants of “Russet Burbank” with resistance to common scab caused by infection with an actinomycete pathogen. Coexpression of resistance to powdery scab caused by a protozoan pathogen was subsequently shown. This study sought to define whether this resistance was effective against additional potato tuber diseases, black scurf, and tuber soft rot induced by fungal and bacterial pathogens. Pot trials and *in vitro* assays with multiple pathogenic strains identified significant resistance to both tuber diseases across the potato variants examined; the best clone A380 showed 51% and 65% reductions in disease severity to tuber soft rot and black scurf, respectively, when compared with the parent line. The resistance appeared to be tuber specific as no enhanced resistance was recorded in stolons or stem material when challenged *Rhizoctonia solani* that induces stolon pruning and stem canker. The work presented here suggests that morphological characteristics associated with tuber resistance may be the predominant change that has resulted from the somaclonal cell selection process, potentially underpinning the demonstrated broad spectrum of resistance to tuber invading pathogens.

## 1. Introduction

Potato (*Solanum tuberosum* L.) tuber diseases black scurf, common scab, powdery scab, and tuber soft rot have both Australian [1–3] and global significance [4] negatively impacting plant growth and tuber quality and quantity. Whilst these diseases may be partly managed with certified seed, crop rotation, agronomic practices, and chemical treatments [4], the development of resistant varieties represents an important disease management option [4].

Russet Burbank is the world's most commonly cropped potato cultivar with yield and quality characteristics that make it the industry standard for the French fry industry sector [5]. Using a novel somaclonal cell selection technique, variants of Russet Burbank were obtained with greatly enhanced resistance to common scab caused by infection with pathogenic *Streptomyces* spp. [3]. Subsequent testing showed these variants coexpressed resistance to powdery scab caused by infection with the protozoan *Spongospora subterranea* subsp. *subterranea* [2]. This breeding strategy aids the retention of key parental traits, compared to the genetic reassortment that occurs with breeding crosses [6]. Expression of resistance to two distinct diseases suggested a possible common mechanism expressed in the tuber periderm. It was postulated that this may confer enhanced resistance to other diverse tuber diseases including black scurf and tuber soft rot.

*Rhizoctonia solani* causes black scurf on potato tubers, stolon and root pruning, and stem canker on above ground plant parts [7]. Black scurf is characterised by the presence of black sclerotia on the tuber surface which result in a significant tuber blemish [7, 8]. Symptoms of stolon and root pruning and stem canker are complex and include sunken brown necrotic lesions on stolons and stems, stolon pruning, delayed emergence, root lesions, and sprout tip damage [8]. *R. solani* has a wide host range and diversity of strains, with anastomosis group (AG)-3 as the predominant potato pathogenic strain [9]. Black scurf is managed using an integrated approach including fungicide [9], certified seed, biological control, and cultural practices [7]. Potato cultivars differ in their susceptibility to *R. solani *[10] although no commercial highly resistant cultivars have been identified or developed [7] despite different approaches, including transgenic [11] and interspecific hybridisations, being attempted [10, 12].

Potato tuber soft rot and blackleg are caused by the bacteria *Pectobacterium atrosepticum (Pa)*,* P. carotovorum *subsp.* carotovorum (Pcc), *and* Dickeya *spp. [13]. The tuber disease, soft rot is initiated through lenticels, meristematic stolons, or wounds [14]. The bacteria produce pectolytic plant cell wall degrading enzymes which allow infiltration and produce macerated tissues that release bacterial inoculum which spreads to other tubers [15]. Rotting can occur in the field or in storage leading to significant losses; the latter is of particular importance to the processing sector where tubers are commonly stored for extended periods prior to factory processing [15]. Blackleg affects the growing plant and is characterised by slimy, black rotting lesions up the stems in wet conditions or stunting, yellowing, and wilting of stems and leaves if conditions are dry [14].

Whilst various breeding strategies have been attempted including hybridisation by somatic fusion with wild diploid species [16] there are no commercial varieties that are naturally immune to soft rot and black leg, although some cultivars show partial resistance [17]. Expression of this resistance to black leg and tuber soft rot is not always associated [18] suggesting that different resistance mechanisms may operate [14] within the tuber and other plant tissues. Multiple disease resistance is an ideal strategy to aid the control of tuber invading pathogens but is rarely achieved. This study sought to define whether the tuber-based resistance to common and powdery scab found in our somaclonal variants of Russet Burbank would be effective against two further diverse tuber diseases. To test this, selected Russet Burbank variants and parental controls were tested against three strains of *Rhizoctonia solani* AG3 and two *Pectobacterium *spp. in glass house trials and *in vitro *assays, respectively.

## 2. Materials and Methods 

### 2.1. Potato (Russet Burbank) Variants Generation and Maintenance

Somaclonal variants of Russet Burbank with enhanced resistance to common scab were obtained using a novel cell selection procedure previously reported [3, 6]. Many of these variants also possessed resistance to powdery scab [2] and showed commercial yield potential equivalent to industry standards [19]. All lines and the parent were maintained as tissue culture plants micropropagated on potato multiplication (PM) medium (MS salts and vitamins [20], plus sucrose (30 g/litre), ascorbic acid (40 mg/litre), casein hydrolysate (500 mg/litre), and 0.8% agar (Sigma-Aldrich, St Louis), with pH adjusted to 5.8). Plants were routinely subcultured as two-node segments every 3 to 4 weeks and incubated at 22°C with a 16 h photoperiod under cool white fluorescent lamps (65 *μ*mol m^−2^ s^−1^). In all resistance screening experiments, four variant clones (A380, TC-RB8, A168a, and A362) and the control parental cultivar Russet Burbank were tested for resistance to tuber soft rot, black scurf, stolon pruning, and stem canker.

### 2.2. Resistance Screening

#### 2.2.1. Tuber Soft Rot *In Vitro* Assays

*Pectobacterium atrosepticum-*DAR30504* (Pa)* and *Pectobacterium carotovorum *subsp. *Carotovorum-*DAR30500 *(Pcc)* were kindly supplied by Orange Agricultural Institute, NSW Government Industry and Investment, NSW, Australia, and maintained on Tryptone Soy Agar (TSA) media. Disease-free tubers of the variants and parent cultivar were surface sterilized (5 min, 0.2% NaOCl) and triple rinsed in sterilized water prior to bruising/wounding treatment. Trials 1 (T1) and 2 (T2) used a bruising method whereby a 1 kg steel weight with a 20 mm head was dropped from 10 cm height onto the centre of the tuber. Trial 3 (T3) used a single site inoculation technique [21] whereby 8 mm sterilized cork borer was used to cut a *c.* 12 mm deep hole in each tuber. After bruising (T1 and T2) or wounding (T3) each tuber was inoculated with the respective pathogen. Inoculation in T1 and T2 involved immersing the bruised tuber in pathogen slurry in water (*c. *10^8^ cells/mL) for 1 minute, whilst in T3 a pathogen suspension in water (*c. *10^8^ cells/mL) was pipetted into the wound hole. After 5 mins individual inoculated tubers were weighed, covered with a plastic wrap, and then placed in a large 50 litre polyethylene bag. All experiments had six tubers (replicates) per treatment. Wet tissue paper was added to the bag to maintain high relative humidity; the bag was tied tightly and incubated in the dark at 20–25°C. After 14 days of incubation the level of rot was assessed by washing tubers to remove rotted tissues and reweighing. The percentage of rotted tissue, a measure of disease severity, was then calculated.

#### 2.2.2. *Rhizoctonia* Pot Trials

Three strains of *Rhizoctonia solani* AG3 were tested separately; strains *R299* and *R422* were kindly supplied by the Department of Primary Industries, Victoria, Australia, and *TAS1* was isolated from surface sterilized sclerotia taken from an infected tuber collected in NW Tasmania, Australia. To prepare inoculum for soil application, 200 gm of millet seed was placed in a conical flask and soaked in 100 mL of water for 1 hour and twice autoclaved for 20 minutes on consecutive days to ensure sterility. Each *R. solani* AG3 strain was then separately transferred to conical flasks and incubated for 14 days (20–22°C) with gentle agitation every two days, to ensure full colonisation.

Three separate glasshouse pot trials (GH1–GH3), one in 2011 with all five variants and two in 2012 with A380, TC-RB8, and the parent only, were undertaken. Four harvest treatments were included: (i) 40, 55, and 70 days after transplanting (stolon and stem disease assessments) and (ii) after senescence (tuber disease assessment). Each clone was replicated in three pots for each harvest treatment and pathogen strain. Plastic pots (20 cm diameter) were filled with pasteurized potting mix containing sand, peat, and composted pine bark (10 : 10 : 80; pH 6.0) premixed with Osmocote 16-3.5-10 NPK resin coated fertiliser (Scotts Australia Pty Ltd.) at the rate of 6 kg/m^3^. In GH1, 10 gm of colonised millet was incorporated into each pot whilst GH2 and GH3 had just 1 gm millet seed (~16 millet seeds) added to each pot. Control pots included sterilized millet seeds. Millet seeds were added to potting soil two weeks before planting and incorporated through the pot by gentle hand mixing.

Planting material was two weeks old; disease–free tissue-cultured plantlets were transplanted into pots (one per pot) and hand watered for the first week. Pots were subsequently watered by overhead sprinklers at 2-day interval, with additional hand irrigation when required to maintain constant wet soil conditions. All trials were undertaken within a glasshouse environment with temperatures maintained at 18 ± 2°C. A soluble fertiliser was applied monthly (Miracle-Gro Water Soluble all-purpose plant food, 15-13.1-12.4 NPK, Scotts Australia Pty Ltd.) and no pesticides were used.

Disease assessments included measures of stolon pruning and stem canker [22] made on three sequential assessment dates, 40, 55, and 70 days after transplanting. Stolon pruning per plant was assessed as follows: 0 = no disease, 1 = 1 or 2 stolons with lesions, 2 = 1 or 2 stolons girdled, 3 = 3 stolons girdled, and 4 = 4 or more stolons girdled. Stem cankering was assessed as an average of all stems from each plant using the rating scale: 0 = no disease, 1 < 10% of stem area covered with lesions, 2 = 10–25% of stem area covered with lesions, 3 = 26–50% of stem area covered with lesions, and 4 = stem area girdled with lesions [22]. Tubers were harvested at plant senescence and washed and each tuber was scored for black scurf according to a visual tuber surface cover score ranging from 0 to 6 (0 = no visible disease on tuber surface, 0.5 ≤ 1%, 1 = 2–5%, 2 = 6–10%, 3 = 11–30%, 4 = 31–50%, 5 = 51–70%, and 6 > 70% of tuber surface affected). The percentage of tuber surface covered by lesions was then estimated by taking the midvalues of these score ranges. The proportion of healthy tubers with no visible lesions was also recorded [23].

### 2.3. Statistical Analysis

*Rhizoctonia *disease data for all tubers per replicate (pot) in all trials were averaged prior to analysis. All data were tested for normality and homogeneity of variance, where appropriate, arcsine transformation was used. Data was then subjected to analysis of variance using GENSTAT v. 14.0 (VSN International Ltd., Hemel Hempstead, UK) with significance calculated at *P* = 0.05, and the least significant difference (LSD) was used for comparison of treatment means.

The estimated percentage surface cover was to illustrate disease severity and was not subject to further statistical comparisons. Mean disease scores (tuber incidence and severity) relative to the control cultivar were generated for each variant by dividing the mean score of each variant by the mean score of the control in each trial in which they were tested and averaging the result.

## 3. Results

### 3.1. Tuber Soft Rot

**Figure 1:**
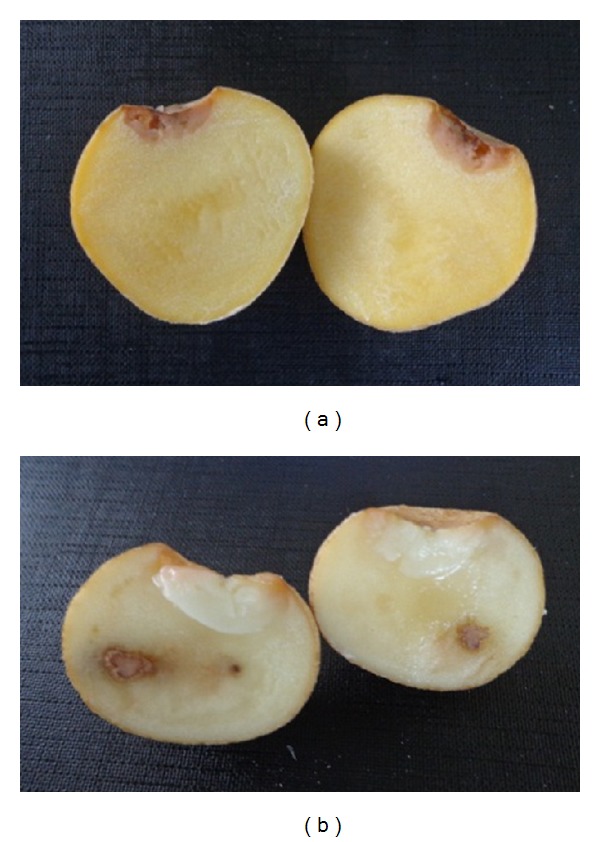
Typical severity of tuber soft rot on potato somaclonal variants (a) A380 and (b) the parent cv. Russet Burbank. Tubers were from *in vitro* trial 1 and the pathogen was *Pectobacterium carotovorum *subsp.* carotovorum (Pcc)*.

Tuber soft rot, as measured through percentage FW of rotten tissue, varied across trials, inoculation technique, and strain of pathogen (Table 1; Figure 1). In trials 1 (T1) and 2 (T2) where bruising treatments were used prior to inoculation, clone A380 had the least rot, significantly less (*P* < 0.05) than the parental control in T1 (*Pcc*) and T2 (both *Pcc* and *Pa*; Figure 1). Variants A168a and A362 (examined in T1 and T2 only) and TC-RB8 also had significantly less rot (*P* < 0.05) than the parent in T1 (A168a and TC-RB8 both against *Pcc*, A362 against *Pa;* Table 1). In trial 3 (T3) where the wounding treatment penetrated the tuber periderm there were no significant differences recorded between the clones A380 and TC-RB8 and the parent (Table 1). The relative mean tuber soft rot severity (average disease severity relative to the parent across all trials where variant was tested) showed that all variants had a reduced (0.49- to 0.76-fold) disease severity, compared to the parental comparator.

### 3.2. Black Scurf

**Figure 2:**
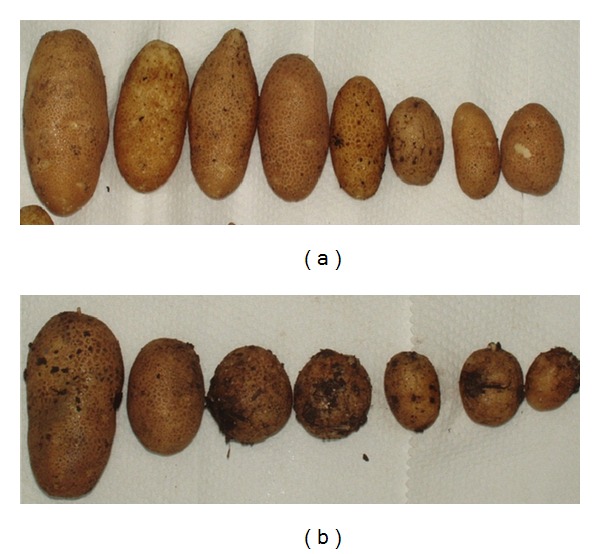
Typical severity of black scurf on potato somaclonal variants (a) A380 and (b) the parent cv. Russet Burbank. Tubers were from glasshouse trial 1 and the pathogen strain *Rhizoctonia solani* AG3 isolate *R299*.

Black scurf disease incidence (Table 2) and severity (Table 3) varied across all trials and between strains of pathogen (Figure 2). Disease incidence was high across all trials with all tubers of the Russet Burbank parent having black scurf symptoms in all trials (Table 2). The variant clones usually had a lower proportion of infected tubers than the parent, although this difference was only significant for A380 in GH3 with isolate *TAS1*. Combining across all trials and strains the variants had 0.65- to 0.89-fold less black scurf incidence than the parent (Table 2). For black scurf severity (Table 3) the Russet Burbank parent had significant greater disease severity (*P* < 0.05) than all tested variant clones when challenged with the least virulent strain (*TAS1*). With the more virulent strains *R299* and *R422* there was a greater variability in results and no significant differences (*P* > 0.05) were recorded; however, similar trends were observed with the parent consistently recording a higher average black scurf severity. Combining across all trials and strains the variants had 0.35- to 0.67-fold less black scurf severity than the parent (Table 3).

### 3.3. Stolon Pruning and Stem Canker

Stolon pruning (Table 4) and stem canker severity (Table 5) showed variability across trials and pathogen isolates. No significant differences were identified between variants when assessing stolon disease (Table 4) and no clear patterns or trends of data were observed, indicating that stolon disease in the somaclones was comparable to that in the parent. In GH1 with strain *R299* variant A362 had significantly more stem canker than both A380 and TC-RB8 but not the parent (Table 5). In all of the other trials, variable data showed no clear trends such that the variants could not be separated from the parental comparator for stem canker severity.

## 4. Discussion

Somaclonal cell selection techniques have been shown to be highly effective in developing extreme resistance against common scab [3] and to provide concurrent resistance against powdery scab [2], two tuber diseases of significance induced by dissimilar pathogens (an actinomycete and a protozoan). Here we demonstrate that these potato variants also possess resistance to additional tuber diseases and diverse pathogen types, tuber soft rot (bacterial infection) and black scurf (fungal infection). Furthermore, resistance expression was shown to operate in the tuber periderm and not in other plant tissues, with no protection against *R. solani *induced stolon pruning and stem canker or soft rot if the tuber periderm was penetrated by the wounding treatment.

Somaclonal selection utilising tissue culture and directed mutagenesis is a rapid breeding strategy that has the benefit of retaining many of the key attributes of the parental line in the derived clones, unlike the genetic reassortment that occurs with traditional breeding sexual crosses [6]. This particularly has importance for the French fry processing sector which has highly specific demands on varietal characteristics. Russet Burbank is over 130 years old [5] and yet remains the most commonly used variety for the French fry processing industry globally despite over a century of breeding efforts. Somaclonal type selection methods have been used to improve resistance to several important potato pathogens [2].

In production of these variants the somaclonal selection process targeted common scab resistance using the pathogen's key pathogenicity determinant, the toxin thaxtomin A, as a positive cell selection agent [6]. The aim was to obtain toxin-tolerant plants that express disease resistance. Strong and robust disease resistance was obtained but not always in variants expressing toxin tolerance [3]. Notwithstanding, thaxtomin A induces scab like symptoms in absence of the pathogen [24]; it stimulates defence responses of the host including electrophysiological [25, 26] and morphological changes including suberisation at the infection site [24]. Given the use of thaxtomin A as a screening agent it can be postulated that the cell selection process may have selected for variants with a rapid suberisation response in the tuber periderm which has led to enhanced broad spectrum disease resistance. There is a growing body of evidence suggesting that increased suberisation and periderm development in the resistance variants may be providing a physical barrier to tuber infection and may be a key structural trait associated with disease resistance [27].

Physical barriers are an important trait in reducing infection of both the tuber soft rot (*Pectobacterium*) and black scurf (*Rhizoctonia*) pathogens. Identification of resistant traits in novel hybrid *Solanum* species has been attributed to a higher degree of esterification of the cell-wall-binding pectin, which is associated with increased structural stability and protection [28], which provides a high level of resistance to black leg and soft rot [16]. Rice varieties resistant to *Rhizoctonia* have a greater amount of cuticular wax on leaf sheaths compared to susceptible varieties [29]. Also, many red-skinned potato varieties with a thinner protective outer periderm layer are more susceptible to pathogen attack than russetted thicker-skinned varieties [30]. Induced production of suberin and antimicrobial agents following pathogen attack is also known to be important in host defence. Susceptible cultivars often produce less and nonuniform deposits of suberin making them more prone to pathogen attack [31, 32].

The relationship between the toxin induced resistance mechanism and actual changes occurring within our somaclonal lines is not determined clearly. However, our physiological evidence from previous studies shows that suberin accumulation in periderm cell layers could be inhibiting disease progression of common scab [27]. The cell selection process is known to create somaclones that can vary morphologically and physiologically [33] and these traits may explain the unique resistance demonstrated against four highly diverse tuber invading pathogens. That somatic cell selection has improved resistance against all these pathogens in a commercially important cultivar, Russet Burbank, demonstrates the importance of this breeding strategy.

## Figures and Tables

**Table 1:** Severity of tuber soft rot in selected potato cv. Russet Burbank variants. Tubers were tested against *Pectobacterium carotovorum *subsp. *carotovorum (Pcc)* and *P. atrosepticum (Pa) *using two different inoculation techniques (T1 and T2—surface bruise; T3—cut) with an incubation period of 14 days.

Variants	Relative tuber disease severity^a^			Disease severity (rotted tissue %)^b^					
				T1 (surface bruise)		T2 (surface bruise)		T3 (cut)	
	Common scab	Powdery scab	Soft rot	*Pcc* ^ c^	*Pa *	*Pcc *	*Pa *	*Pcc *	*Pa *
A380	0.19	0.44	0.49	7.22^a^	9.03^a^	9.78	5.37^a^	23.71	2.37
A168a	0.21	0.36	0.71	20.44^b^	18.15^abc^	14.2	22.00^b^	—	—
TC-RB8	0.57	0.98	0.76	15.60^ab^	23.29^bc^	21.80	12.89^ab^	16.78	3.17
A362	0.66	0.80	0.73	26.66^bc^	12.61^ab^	20.62	14.97^ab^	—	—
Russet Burbank (parent)	1.00	1.00	1.00	34.21^c^	29.63^c^	19.02	24.42^b^	21.68	3.65
*P*				<0.001	0.006	0.229	0.007	0.441	0.331
LSD (0.05)			12.09	12.88	ns	10.82	ns	ns

—: lines not tested within the specified trial.

^a^Mean relative disease severity scores for common scab, powdery scab, and tuber soft rot were generated for each variant by dividing the mean disease score of each variant by the mean disease score of the parent cultivar in each trial in which they appeared and averaging the result. Common scab data are from [3] and powdery scab data are from [2].

^b^Percentage of rotten tissues from individual tuber was calculated (*n* = 12).

^c^Means followed by the same letter within the same column are not significantly different at *P* = 0.05 using Fisher's LSD test; ns: nonsignificant.

**Table 2:** Incidence of black scurf in selected potato cv. Russet Burbank variants in glasshouse (GH1—2010; GH2 and GH3—2011) trials. *Rhizoctonia solani* AG3 isolates *R299, R422*, and* TAS1 *were tested separately in each trial.

Variants	Relative tuber disease incidence^a^			Disease incidence (infected tubers (%))^b^								
				GH1			GH2			GH3		
	Common scab	Powdery scab	Black scurf	*R299 *	*R422 *	*TAS1 *	*R299 *	*R422 *	*TAS1 *	*R299 *	*R422 *	*TAS1* ^ c^
A380	0.27	0.53	0.65	71.3 (1.09)	100 (1.57)	64.2 (0.85)	66.7 (0.87)	61.1 (0.66)	66.7 (0.88)	77.8 (1.16)	44.4 (0.64)	36.7 (0.39)^a^
A168a	0.32	0.49	0.89	100 (1.57)	100 (1.57)	67.5 (0.97)	—	—	—	—	—	—
TC-RB8	0.66	0.99	0.86	98.6 (1.48)	100 (1.57)	61.7 (0.82)	83.3 (1.22)	69.4 (0.92)	72.2 (0.94)	100 (1.57)	100 (1.57)	86.7 (1.26)^ab^
A362	0.78	0.80	0.86	100 (1.57)	91.4 (1.32)	66.1 (0.90)	—	—	—	—	—	—
Russet Burbank (parent)	1.00	1.00	1.00	100 (1.57)	100 (1.57)	100 (1.57)	100 (1.57)	100 (1.57)	100 (1.57)	100 (1.57)	100 (1.57)	100.0 (1.57)^b^
*P*				0.12	0.06	0.17	0.44	0.07	0.34	0.44	0.12	0.04
LSD (0.05)			ns	ns	ns	ns	ns	ns	ns	ns	0.89

—: lines not tested within the specified trial.

^a^Mean relative disease incidence for common scab, powdery scab, and black scurf was generated for each variant by dividing the mean disease score of each variant by the mean disease score of the parent cultivar in each trial in which they appeared and averaging the result. Common scab data are from [3] and powdery scab data are from [2].

^b^Data are mean percentage of tubers with any black scurf symptoms and transformed arcsine means (in parentheses).

^c^Transformed means followed by the same letter within the same column are not significantly different at *P* = 0.05 using Fisher's LSD test; ns: non-significant.

**Table 3:** Severity of black scurf in selected potato cv. Russet Burbank variants in glasshouse (GH1—2010; GH2 and GH3—2011) trials. *Rhizoctonia solani* AG3 isolates *R299, R422*, and* TAS1 *were tested separately in each trial.

Variants	Relative tuber disease severity^a^			Disease severity (surface cover score 0–6 (%))^b^								
				GH1			GH2			GH3		
	Common scab	Powdery scab	Black scurf	*R299 *	*R422 *	*TAS1* ^ c^	*R299 *	*R422 *	*TAS1 *	*R299 *	*R422 *	*TAS1 *
A380	0.19	0.44	0.35	2.17 (19.5)	1.90 (9.8)	0.5^b^ (1.22)	0.47 (0.97)	0.58 (1.64)	0.33^b^ (.33)	0.56 (1.22)	0.33 (0.78)	0.18^b^ (0.18)
A168a	0.21	0.36	0.67	3.42 (34.2)	1.45 (6.3)	0.71^b^ (2.48)	—	—	—	—	—	—
TC-RB8	0.57	0.98	0.54	3.25 (27.9)	1.42 (7.2)	0.99^b^ (5.05)	1.01 (3.80)	0.44 (0.83)	0.53^b^ (1.19)	0.85 (2.18)	1.10 (3.99)	0.50^b^ (0.77)
A362	0.66	0.8	0.66	2.93 (22.3)	1.27 (5.8)	0.97^b^ (4.41)	—	—	—	—	—	—
Russet Burbank (parent)	1	1	1	3.15 (26.4)	2.35 (16.8)	2.17^a^ (14.18)	2.17 (12.67)	1.76 (13.07)	1.66^a^ (7.18)	1.77 (9.63)	1.17 (3.75)	1.67^a^ (7.33)
*P *				0.39	0.22	0.04	0.12	0.25	0.01	0.14	0.16	0.01
LSD (0.05)				ns	ns	1.11	ns	ns	0.75	ns	ns	0.76

—: lines not tested within the specified trial.

^a^Mean relative disease severity score for common scab, powdery scab, and black scurf was generated for each variant by dividing the mean disease score of each variant by the mean disease score of the parent cultivar in each trial in which they appeared and averaging the result. Common scab data are from [3] and powdery scab data are from [2].

^b^Tuber disease surface cover score—0: no disease, 0.5 ≤ 1%, 1: 2–5%, 2: 6–10%, 3: 11–30%, 4: 31–50%, 5: 51–70%, and 6 > 70%. In brackets: estimated tuber surface coverage is calculated from disease cover score using median percentile scores within the allocated range.

^c^Means followed by the same letter within the same column are not significantly different at *P* = 0.05 using Fisher's LSD test; ns: non-significant.

**Table 4:** Susceptibility to *Rhizoctonia* stolon pruning of selected potato cv. Russet Burbank variants in glasshouse (GH1—2010; GH2 and GH3—2011) trials. *Rhizoctonia solani* AG3 isolates *R299, R422*, and* TAS1 *were tested separately in each trial.

Variants	Mean stolon pruning^a^—GH1			Mean stolon pruning—GH2			Mean stolon pruning—GH3		
	*R299* ^ b^	*R422 *	*TAS1 *	*R299 *	*R422 *	*TAS1 *	*R299 *	*R422 *	*TAS1 *
A380	0.75	0.88	1.25	0.33	0.00	0.33	0.67	0.33	0.33
A168a	2.88	0.88	1.63	—	—	—	—	—	—
TC-RB8	2.00	1.25	0.75	0.00	0.00	0.00	0.33	0.67	1.00
A362	2.50	1.88	2.13	—	—	—	—	—	—
Russet Burbank (parent)	1.50	0.88	1.38	0.33	0.00	0.00	0.67	1.00	1.00
*P*	0.11	0.47	0.56	0.69	—	0.44	0.79	0.44	0.11
LSD (0.05)	ns	ns	ns	ns	ns	ns	ns	ns	ns

—: lines not tested within the specified trial.

^a^Stolon pruning disease score—0: no disease, 1: 1 or 2 stolons with lesions, 2: 1 or 2 stolons girdled, 3: 3 stolons girdled, and 4: 4 or more stolons girdled.

^b^Means followed by the same letter within the same column are not significantly different at *P* = 0.05 using Fisher's LSD test; ns: non-significant.

**Table 5:** Stem canker severity evaluation of selected potato cv. Russet Burbank variants in glasshouse (GH1—2010; GH2 and GH3—2011) trials. *Rhizoctonia solani* AG3 isolates *R299, R422*, and* TAS1 *were tested separately in each trial.

Variants	Mean stem canker^a^—GH1			Mean stem canker—GH2			Mean stem canker—GH3		
	*R299* ^ b^	*R422 *	*TAS1 *	*R299 *	*R422 *	*TAS1 *	*R299 *	*R422 *	*TAS1 *
A380	0.63^a^	0.06	0.06	0.67	0.67	0.67	0.00	0.33	0.33
A168a	0.94^ab^	0.63	0.44	—	—	—	—	—	—
TC-RB8	0.31^a^	0.38	0.25	1.33	0.67	0.00	0.67	1.00	0.00
A362	1.63^b^	0.94	0.56	—	—	—	—	—	—
Russet Burbank (parent)	0.88^ab^	0.63	0.63	0.33	0.50	0.00	0.67	0.67	0.33
*P*	0.02	0.40	0.149	0.28	0.91	0.11	0.31	0.25	0.69
LSD (0.05)	0.80	ns	ns	ns	ns	ns	ns	ns	ns

—: lines not tested within the specified trial.

^a^Stem canker disease score—0: no disease, 1: <10% of stem area covered with lesions, 2: 10–25% of stem area covered with lesions, 3: 26–50% of stem area covered with lesions, and 4: stem girdled with lesions.

^b^Means followed by the same letter within the same column are not significantly different at *P* = 0.05 using Fisher's LSD test; ns: nonsignificant.
